# Presenilins regulate synaptic plasticity in the perforant pathways of the hippocampus

**DOI:** 10.1186/s13041-023-01009-x

**Published:** 2023-01-30

**Authors:** Sang Hun Lee, Vadim Y. Bolshakov, Jie Shen

**Affiliations:** 1 Department of Neurology, Brigham & Women’s Hospital, Harvard Medical School, Boston, MA 02115 USA; 2 Department of Psychiatry, McLean Hospital, Harvard Medical School, Belmont, MA 02478 USA; 3 Program in Neuroscience, Harvard Medical School, Boston, MA 02115 USA

**Keywords:** Alzheimer’s disease, Conditional knockout, Hippocampus, Perforant pathways, Short-term synaptic plasticity, Long-term potentiation, Calcium homeostasis

## Abstract

Mutations in the *Presenilin* genes (*PSEN1* and *PSEN2*) are the major cause of familial Alzheimer’s disease (AD), highlighting the importance of Presenilin (PS) in AD pathogenesis. Previous studies of PS function in the hippocampus demonstrated that loss of PS results in the impairment of short- and long-term synaptic plasticity and neurotransmitter release at hippocampal Schaffer collateral (SC) and mossy fiber (MF) synapses. Cortical input to the hippocampus through the lateral perforant pathway (LPP) and the medial perforant pathway (MPP) is critical for normal cognitive functions and is particularly vulnerable during aging and early stages of AD. Whether PS regulates synaptic function in the perforant pathways, however, remained unknown. In the current study, we investigate PS function in the LPP and MPP by performing whole-cell and field-potential electrophysiological recordings using acute hippocampal slices from postnatal forebrain-restricted excitatory neuron-specific *PS* conditional double knockout (cDKO) mice. We found that paired-pulse ratio (PPR) is reduced in the LPP and MPP of *PS* cDKO mice. Moreover, synaptic frequency facilitation or depression in the LPP or MPP, respectively, is impaired in *PS* cDKO mice. Notably, depletion of intracellular Ca^2+^ stores by inhibition of sarcoendoplasmic reticulum Ca^2+^ ATPase (SERCA) minics and occludes the effects of PS inactivation, as evidenced by decreases of the evoked excitatory postsynaptic currents (EPSCs) amplitude in the LPP and MPP of control neurons but no effect on the EPSC amplitude in *PS* cDKO neurons, suggesting that impaired intracellular calcium homeostasis in the absence of PS may contribute to the observed deficits in synaptic transmission. While spontaneous synaptic events, such as both the frequency and the amplitude of spontaneous or miniature EPSCs, are similar between *PS* cDKO and control neurons, long-term potentiation (LTP) is impaired in the LPP and MPP of *PS* cDKO mice, accompanied with reduction of evoked NMDA receptor-mediated responses. These findings show the importance of PS in the regulation of synaptic plasticity and intracellular calcium homeostasis in the hippocampal perforant pathways.

## Introduction

Alzheimer’s disease (AD) is the most common neurodegenerative disorder characterized by progressive memory loss and cognitive decline. Mutations in the *Presenilin* genes (*PSEN1* and *PSEN2*) account for > 80% of all identified causative mutations in familial AD, highlighting the importance of Presenilin (PS) in AD pathogenesis. Previous genetic studies demonstrated that PS plays essential roles in the developing brain and in the adult cerebral cortex [1–12]. Inactivation of PS in excitatory neurons of the postnatal forebrain results in deficits in short-term synaptic plasticity, long-term potentiation (LTP), NMDA receptor function, neurotransmitter release, and calcium homeostasis [4, 7–9, 11]. Interestingly, the presynaptic defects occur prior to the postsynaptic deficits in postnatal forebrain-restricted, excitatory-specific *PS* conditional double knockout (cDKO) mice [8]. Furthermore, inactivation of PS in hippocampal CA3 neurons, but not CA1 neurons, results in impaired LTP, paired pulse facilitation and frequency facilitation in the Schaffer collateral (SC) pathway, and presynaptic PS regulates neurotransmitter release and LTP via modulation of ryanodine receptor (RyR) levels and RyR-mediated calcium release from the endoplasmic reticulum (ER) [7, 9].

The entorhinal cortex (EC) is the major input and output structure of the hippocampal formation, forming the nodal point in cortico-hippocampal circuits [13]. Specifically, EC layer II stellate neurons projecting to dentate gyrus (DG) through the perforant pathways form glutamatergic synapses on DG granule neurons but with different functional properties depending on where the afferents are coming from: either from the lateral perforant pathway (LPP) or the medial perforant pathway (MPP) [14]. Indeed, MPP presynaptic terminals have a higher vesicle release probability than those of the LPP [15–17]. Importantly, cortical input to the hippocampus through perforant pathways is critical for normal cognitive function but is particularly vulnerable during aging and at early stages of AD. Previous analysis of AD brains showed accumulation of amyloid plaques and neurofibrillary tangles both in the EC and the molecular layer of the DG [18, 19], massive loss of entorhinal layer II neurons [20, 21], and decreased number of synapses in the outer molecular layer of the DG [22, 23]. These findings suggest that AD-related pathologic alterations impact both the EC and the hippocampal formation. Despite the importance of EC-hippocampal network, the role of PS in the hippocampal perforant pathways have not been addressed. Furthermore, despite extensive evidence indicating that PS regulates synaptic functions in hippocampal SC and mossy fiber (MF) pathways [3, 4, 7–12], it is unknown whether PS plays a similar or distinct role in the regulation of synaptic function in the hippocampal perforant pathways.

In the present study, we investigate the role of PS in the hippocampal LPP and MPP using our previous developed postnatal forebrain-restricted, excitatory neuron-specific *PS* cDKO mice at 2 months of age, before the onset of neurodegeneration [4, 24]. We found that paired-pulse ratio (PPR) and synaptic facilitation or depression induced by frequent stimulation are impaired in both LPP and MPP of *PS* cDKO mice. The increase or decrease of the evoked EPSC amplitude in the course of repetitive presynaptic stimulation of the LPP or MPP, respectively, is impaired in *PS* cDKO mice, and blockade of SERCA mimics and occludes the synaptic deficits in *PS* cDKO neurons. While basal synaptic transmission is unaffected, LTP and NMDA receptor-mediated responses are reduced at both LPP and MPP synapses of *PS* cDKO mice. Taken together, our study demonstrates the importance of PS in the regulation of synaptic plasticity and Ca^2+^ homeostasis at hippocampal LPP and MPP synapses.

## Methods

### Mice

The generation and extensive characterization of postnatal forebrain-restricted excitatory neuron-specific *PS* cDKO mice were previously reported [3, 4, 7, 8, 24, 25]. Northern, in situ hybridization, and Western analyses were carried out to confirm normal PS1 expression in control (*fPS1/fPS1*) mice and inactivation of PS1 in the cerebral cortex of *PS* cDKO (*fPS1/fPS1; PS2*^*−/−*^*; αCaMKII-Cre*) mice beginning at postnatal day ~ 18 and complete at ~ 4 weeks of age [4, 7–9, 11, 24]. All mice used in the current study were housed in humidity- and temperature-controlled rooms maintained on a 12:12 h light: dark cycle and were given standard rodent chow and water. *PS* cDKO and control mice were maintained in the C57BL/6J 129 hybrid genetic background. All procedures were approved by the IACUC committees of Brigham and Women’s Hospital, and conform to the USDA Animal Welfare Act, PHS Policy on Humane Care and Use of Laboratory Animals, the “ILAR Guide for the Care and Use of Laboratory Animals” and other applicable laws and regulations.

### Preparation of brain slices for electrophysiology

Hippocampal slices were prepared from both male and female *PS* cDKO and control mice at 2 months of age. Mice were decapitated after being anesthetized with ketamine (100 mg/kg) + xylazine (10 mg/kg) + acepromazine (3 mg/kg). The brain was removed and placed in ice-cold (4 °C) oxygenated (95% O_2_/5% CO_2_) high sucrose and magnesium solution containing (in mM) the following: 200 Sucrose, 25 NaHCO_3_, 10 Glucose, 3 KCl, 1.25 NaH_2_PO_4_, 1.2 Na-pyruvate and 0.4 Na-ascorbate, 7 MgCl_2_, and 0.5 CaCl_2_. Horizontal hippocampal slices (400 μm thick) were prepared using a vibratome (VT1200S, Leica, Germany), and transferred to an incubation chamber filled with oxygenated artificial cerebrospinal fluid (ACSF) containing (in mM) the following: 125 NaCl, 3 KCl, 1.25 NaH_2_PO_4_, 1 MgCl_2_, 2 CaCl_2_, 25 NaHCO_3_, 10 Glucose, 1.2 Na-pyruvate and 0.4 Na-ascorbate, adjusted to 310 ± 5 mOsm (pH 7.4). The slices were allowed to recover at 34 °C for 1 h and then placed in a recording chamber constantly perfused with heated ACSF (30 ± 1 °C) and gassed continuously with 95% O_2_ and 5% CO_2_. The flow rate of bathing solution and the volume of the recording chamber for slices were 2.2 ml/min and 1.2 ml, respectively. Hippocampal slices were observed using an upright microscope equipped with differential interference contrast (DIC) optics (BX51WI, Olympus, Japan). The DIC optics was used for visualization of neurons in the course of whole-cell recordings. In a subset of experiments, the following drugs were used at the following concentrations via bath application or adding to intracellular recording solution: Picrotoxin (100 µM, Tocris #1128), Bicuculline methochloride (20 µM, Tocris #0131), D-AP5 (50 µM, Tocris #0106), NBQX disodium salt (10 μM, Tocris #1044), QX314 chloride (5 mM, Tocris #2313) and Thapsigargin (2 μM, Sigma-Aldrich #T9033).

### Electrophysiological analysis

For extracellular field potential recordings, stimulation pulses were delivered with a stimulus isolation unit (A385, World Precision Instruments, USA) using the unipolar metal stimulation microelectrode. Field excitatory postsynaptic potentials (fEPSPs) were recorded in current-clamp mode with ACSF-filled patch pipettes (1.5–2 MΩ). All fEPSPs were recorded with a stimulation strength that yielded ~ 50% of the maximal response. Data were collected with a MultiClamp 700B amplifier (Molecular Devices, USA) and digitized at 10 kHz using the A/D converter DIGIDATA 1322A (Molecular Devices, USA). Data were acquired and analyzed using a custom program written with Igor Pro software (Version 6.3; Wave-Metrics) and Clampfit (Version 10.3; Molecular device).

For AMPA receptor-mediated input/output (I/O) curves, I/O relations were obtained by plotting the amplitude of fiber volley (FV) versus the fEPSP slope in the presence of blockers of NMDA (50 µM AP5) and GABA_A_ receptors (100 µM Picrotoxin). 10 traces were averaged for each stimulation intensity, and the amplitude of the FV was measured relative to the slope of the fEPSP. The stimulation rate was 0.2 Hz. The average linear fit slope was calculated to obtain I/O relationships for each slice tested. In LTP recordings, after baseline responses were collected every 15 s for 15 min, LTP was induced by five episodes of theta burst stimulation (TBS) delivered at 0.1 Hz. Each episode contained ten stimulus trains (5 pulses at 100 Hz) delivered at 5 Hz. To generate summary graphs (mean ± SEM), individual experiments were normalized to the baseline, and four consecutive responses were averaged to generate 1 min bins. These were then averaged together to generate the final summary graphs. Paired-pulse facilitation (PPF) was measured as the ratio of the second fEPSP slope relative to the first fEPSP slope, evoked by two identical presynaptic stimuli. Synaptic facilitation was measured as the percentage of the second fEPSP slope versus the first fEPSP slope at a given stimulus train in individual slices.

For whole-cell patch clamp experiments, recording pipettes (3–5 MΩ) were filled with a solution containing (in mM) the following: 120 K-gluconate, 10 KCl, 20 HEPES, 4 MgATP, 0.3 NaGTP, 10 phosphocreatine, and 0.2 EGTA with the pH adjusted to 7.30 with KOH (295–300 mOsm). Spontaneous or miniature excitatory postsynaptic currents (sEPSCs or mEPSCs) were recorded from dentate gyrus granule neurons in voltage-clamp mode at a holding potential of −80 mV in the presence of 100 µM picrotoxin for the blockade of GABA_A_ receptor without or with 1 µM TTX, respectively. To obtain the NMDA/AMPA EPSC amplitude ratio, the evoked AMPA receptor- and NMDA receptor-mediated EPSCs were recorded in voltage clamp mode in the same cell at −80 mV and +40 mV in the presence of blocker of GABA_A_ receptor (10 µM bicuculline), respectively. The NMDA receptor-mediated component of the EPSC was measured 50 ms after the peak of the AMPA receptor EPSCs. The intracellular solution in these experiments contained (in mM) the following: 120 Cs-methanesulfonate, 20 tetraethylammonium-chloride, 20 HEPES, 4 MgATP, and 0.3 NaGTP, 5 QX314 and 0.2 EGTA with the pH adjusted to 7.30 with CsOH (295–300 mOsm). The series resistance (Rs) after establishing whole-cell configuration was between 15 and 25 MΩ. EPSC recordings with > 20% series resistance changes were excluded from the data analysis.

### Data quantification and statistical analysis

Data acquisition and quantification were performed in a genotype blind manner. All statistical analysis was performed using Prism (Version 9; GraphPad software), Excel (Microsoft), Igor Pro (Version 6.3; Wave-Metrics) or Clampfit (Version 10.3; Molecular device). All data are presented as the mean ± SEM. The exact sample size (e.g., the number of mice, brain slices or neurons) of each experiment is indicated in the figure.

**Fig. 1:**
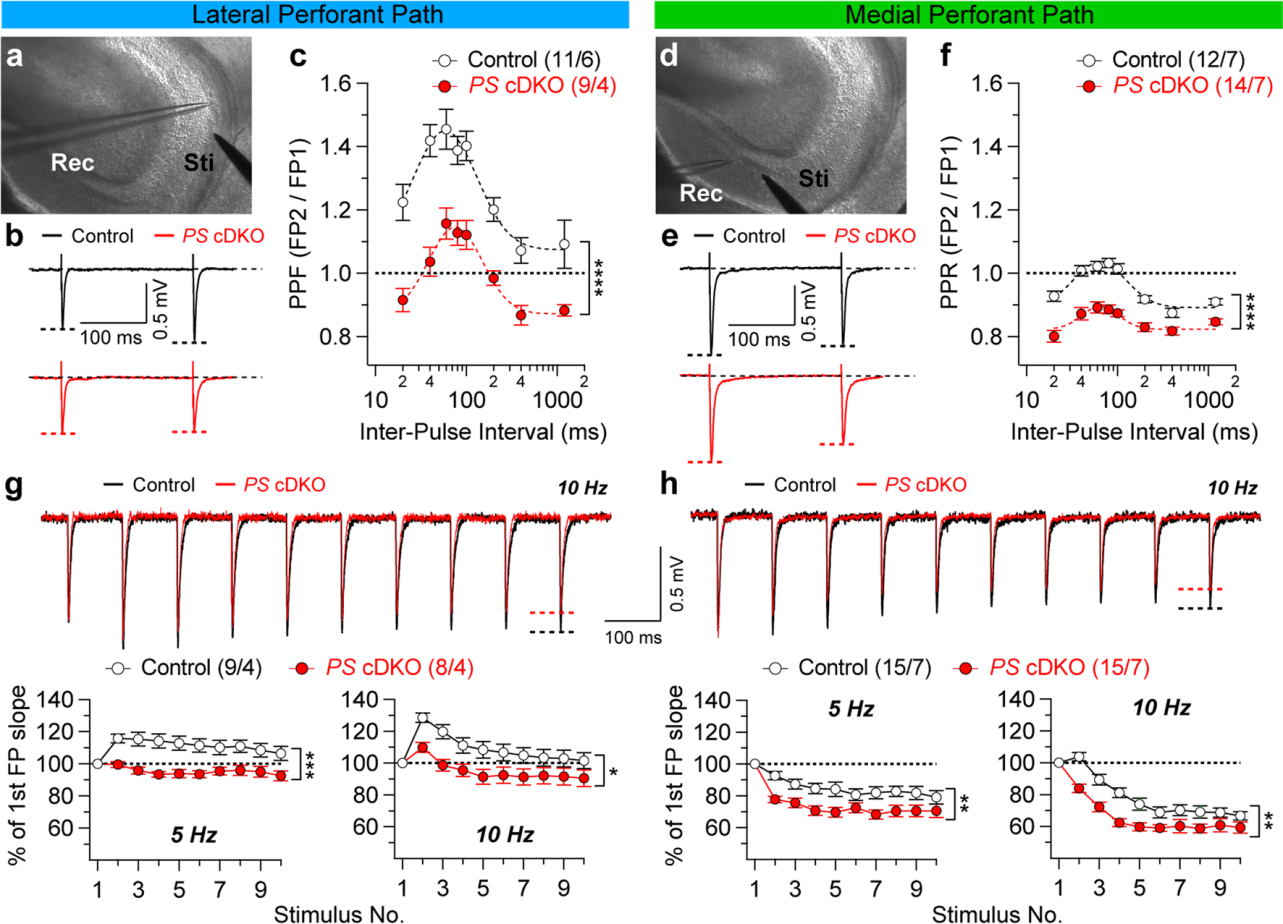
Impaired short-term plasticity in lateral and medial perforant pathways of *PS* cDKO mice. **a** and **d** DIC images of DG area in an acute hippocampal slice in which the stimulation (Sti) and recording pipettes (Rec) are positioned at the outer or inner layer of molecular region to stimulate lateral or medial perforant pathway, respectively. **b** and **e** Representative traces of fEPSPs evoked by two consecutive stimuli with a 200 ms inter-stimulus interval. **c** and **f** Average paired-pulse ratio (PPR) values plotted as a function of the inter-stimulus interval (20–1200 ms) show reduced PPR in *PS* cDKO mice (F_1, 18_ = 36.19, p < 0.0001 in LPP; F_1, 24_ = 48.26; p < 0.0001 in MPP; two-way ANOVA). **g** and **h** Top: Superimposed fEPSP traces of frequency facilitation (g) or depression (h) elicited by 10 Hz stimulus trains show significant reduction in *PS* cDKO mice relative to control mice. Bottom: Summary graphs show that synaptic facilitation or depression elicited by stimulus trains are impaired in both LPP and MPP of *PS* cDKO mice (LPP at 5 Hz: F_1, 15_ = 22.47, p = 0.0003; LPP at 10 Hz: F_1, 15_ = 7.96, p = 0.0129; MPP at 5 Hz: F_1, 28_ = 8.29, p = 0.0075; MPP at 10 Hz: F_1, 28_ = 12.97, p = 0.0012; two-way ANOVA). The slopes of shown fEPSP are normalized to the slope of the first fEPSP of the stimulus train. All data represent mean ± SEM (*p < 0.05, **p < 0.01, ***p < 0.001, ****p < 0.0001). The number of slices/mice used in each experiment is shown in parentheses

**Fig. 2:**
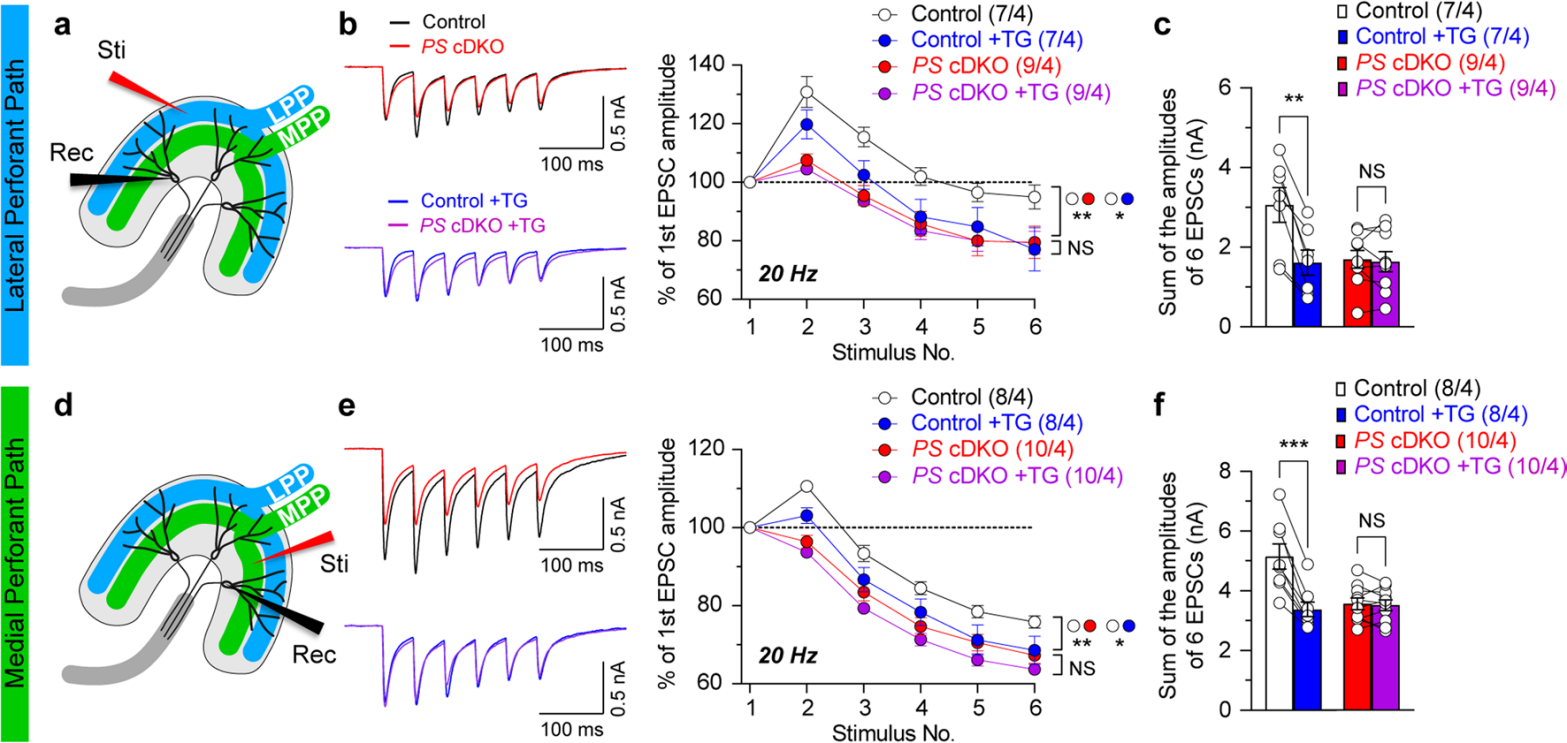
Impairment of ER Ca^2+^ homeostasis in lateral and medial perforant pathways of *PS* cDKO mice. **a** and **d** Depicted images of DG area show that the stimulation (Sti) and recording pipette (Rec) are positioned at the LPP or MPP, respectively. **b** Representative EPSCs evoked by 6 repetitive LPP stimuli at 20 Hz recorded before and after addition of the SERCA inhibitor, TG (2 μM for 30 min). The evoked EPSCs were elicited with 100 µA stimulation intensity by the stimulation electrode placed at LPP and recorded at a holding potential of −80 mV in whole-cell configuration. Summary graphs show that the frequency facilitation of evoked EPSCs is decreased in *PS* cDKO neurons relative to slices from control mice (p = 0.0025; two-way ANOVA). The TG treatment of slices from control mice results in reduced frequency facilitation of EPSC amplitudes (p = 0.04; two-way ANOVA), whereas TG treatment of slices from *PS* cDKO mice fails to reduce the magnitude of frequency facilitation triggered by stimulus trains (p = 0.75; two-way ANOVA). **c** Summary graphs display the sum of EPSC amplitudes before and after TG application at LPP. **e** Representative EPSCs evoked by 6 repetitive MPP stimuli at 20 Hz recorded before and after addition of TG. Frequency depression of evoked EPSCs elicited by the MPP stimulation becomes more pronounced in slices from *PS* cDKO mice (p = 0.0012, two-way ANOVA). The TG treatment of slices from control mice results in enhanced frequency depression (p = 0.04, two-way ANOVA), whereas TG treatment of slices from *PS* cDKO mice fails to further enhance frequency depression (p = 0.14; two-way ANOVA). **f** Summary graphs display the sum of EPSC amplitudes before and after TG application at MPP. All data represent mean ± SEM (*p < 0.05, **p < 0.01, ***p < 0.001; NS: not significant). The number of neurons/mice used in each experiment is shown in parentheses

**Fig. 3:**
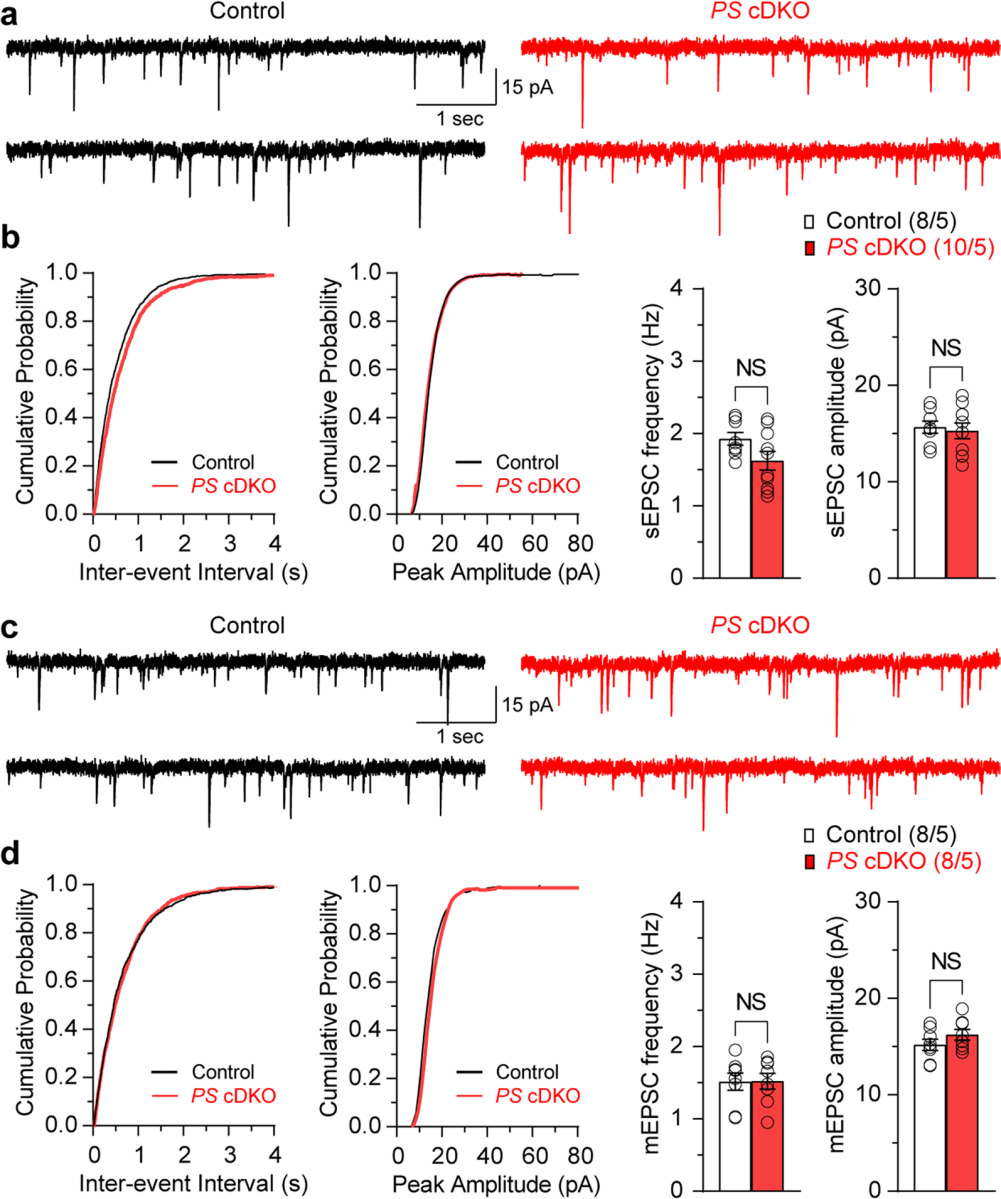
Normal spontaneous and miniature EPSCs in *PS* cDKO mice. **a** Representative sEPSCs recorded in dentate gyrus granule neurons from control and *PS* cDKO mice at a holding potential of −80 mV in the presence of blocker of GABA_A_ receptor (100 µM picrotoxin). **b** Statistical analysis indicates normal sEPSC frequency (p = 0.083, unpaired *t*-test) and amplitude (p = 0.725, unpaired *t*-test) in *PS* cDKO neurons. **c** Representative mEPSCs recorded in dentate gyrus granule neurons from control and *PS* cDKO mice at a holding potential of −80 mV in the presence of 100 µM picrotoxin for the blockade of GABA_A_ receptor with 1 µM TTX. **d** Statistical analysis indicates similar mEPSC frequency (p = 0.967, unpaired *t*-test) and amplitude (p = 0.216, unpaired *t*-test) between *PS* cDKO and control neurons. All data represent mean ± SEM (*NS* not significant). The number of neurons/mice used in each experiment is shown in parentheses

**Fig. 4:**
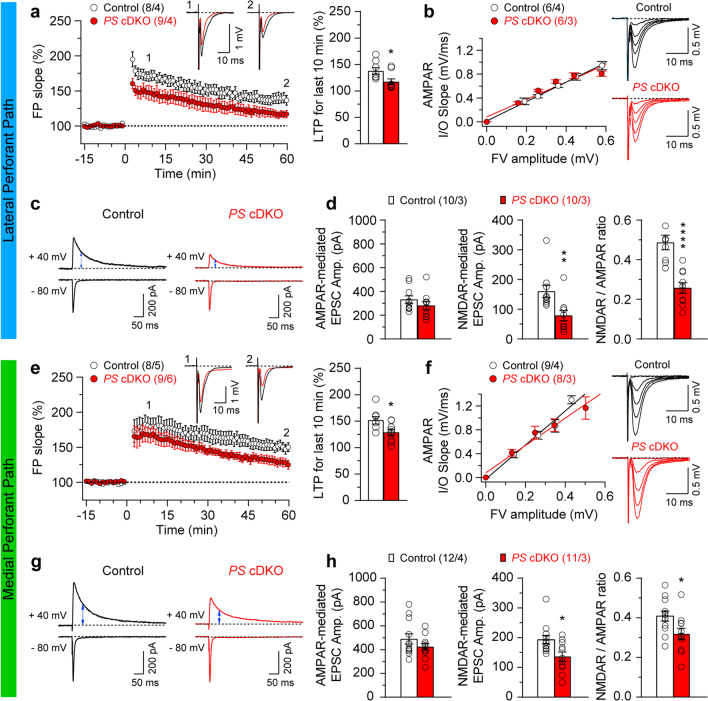
Impaired LTP and NMDA receptor activation in LPP and MPP of *PS* cDKO mice. **a** and **e** Impaired LTP induced by 5 TBS in LPP and MPP of *PS* cDKO mice. Superimposed traces are averages of four consecutive responses 7 min and 60 min after (1, 2) TBS induction. **b** and **f** Normal AMPAR-mediated input/output curves in both lateral (Control: y = 1.63x, R^2^ = 0.90; *PS* cDKO: y = 1.46x, R^2^ = 0.88) and medial (Control: y = 2.86x, R^2^ = 0.83; *PS* cDKO: y = 2.29x, R^2^ = 0.63) perforant pathway of *PS* cDKO mice at 2 months of age. The lines represent the best linear regression fit. The input/output slopes are similar between control and *PS* cDKO mice (LPP: p = 0.22; MPP: p = 0.11; linear regression). **c** and **g** The sample traces of evoked AMPAR- and NMDAR-mediated EPSCs recorded in whole-cell voltage clamp mode in the same cell at −80 mV (lower traces) and +40 mV (upper traces) in the presence of Bicuculline, respectively, are shown. The NMDAR-mediated component of the EPSC was measured 50 ms after the peak of the AMPAR EPSCs (marked with blue arrow heads). **d** and **h** Summary graphs from left to right show AMPAR- or NMDAR-mediated EPSC amplitudes and NMDAR/AMPAR ratios. Note that the NMDAR/AMPAR ratio is reduced in both LPP and MPP of *PS* cDKO neurons (LPP: Control: 0.49 ± 0.04, *PS* cDKO: 0.26 ± 0.03, p < 0.0001; MPP: Control: 0.41 ± 0.03, *PS* cDKO: 0.32 ± 0.03, p = 0.028; unpaired *t*-test). All data represent means ± SEM (*p < 0.05, **p < 0.01, ****p < 0.0001). The number of slices or neurons/mice used in each experiment is shown in parentheses

Statistical analysis was conducted using Student’s *t*-test (Figs. 2c, f, 3b, d, 4a, d, e, h) or two-way ANOVA followed up by Bonferroni multiple comparison test (Figs. 1c, f, g, h, 2b, 2e) and linear regression fit (Fig. 4b, 4f). All statistical comparisons were performed on data from ≥ 3 biologically independent samples and replicated on different experimental days. Significance is shown as *p < 0.05, **p < 0.01, ***p < 0.001, ****p < 0.0001, or NS (not significant).

## Results

### Impaired short-term plasticity in the hippocampal perforant pathways in *PS* cDKO mice

The perforant pathways, originating from pyramidal neurons in layer II of the EC, are divided into lateral perforant path (LPP) and medial perforant path (MPP). Each pathway has different anatomical and functional properties depending on the afferents in either of two mentioned projections [14]. In our experiments, the LPP or MPP were activated selectively by carefully positioning electrodes in the lateral one-third or medial molecular layer of the dentate gyrus supra-pyramidal blade (to activate LPP or MPP, respectively; Fig. 1a and d). Distinguishing LPP and MPP inputs primarily relied on the use of electrophysiological paired-pulse stimulation protocol. The administration of paired-pulse stimuli typically results in the second pulse being facilitated when the LPP is activated but paired-pulse depression when the MPP is stimulated [15, 16, 26, 27].

To determine whether the loss of PS affects synaptic function at LPP or MPP synapses, we assayed short-term plasticity in the excitatory neuron-specific *PS* cDKO mice at 2 months of age. We found that paired-pulse ratio (PPR) is significantly reduced at both LPP and MPP synapses in *PS* cDKO mice relative to controls, indicating an impairment of short-term plasticity at the perforant pathways in the absence of PS (Fig. 1c and f; LPP: F_1, 18_ = 36.19, p < 0.0001; MPP: F_1, 24_ = 48.26; p < 0.0001; two-way ANOVA). Consistent with these results, frequency facilitation of synaptic responses induced by short trains of presynaptic stimulation (10 pulses), which were delivered at the frequencies of 5 and 10 Hz, is also significantly reduced in the LPP of *PS* cDKO mice (Fig. 1g; 5 Hz: F_1, 15_ = 22.47, p = 0.0003; 10 Hz: F_1, 15_ = 7.96, p = 0.0129), whereas frequency depression was enhanced in the MPP of *PS* cDKO mice (Fig. 1h; 5 Hz: F_1, 28_ = 8.29, p = 0.0075; 10 Hz: F_1, 28_ = 12.97, p = 0.0012; two-way ANOVA).

These results, together with our earlier findings at SC and MF synapses [4, 7–9, 11], further demonstrate that PS plays an essential and universal role in the regulation of short-term synaptic plasticity thus possibly contributing to the regulation of neurotransmission in the hippocampus.

### Impaired ER Ca^2+^ homeostasis in the hippocampal perforant pathways in *PS* cDKO mice

To determine whether the reduction of short-term synaptic plasticity in both LPP and MPP inputs in *PS* cDKO mice might be due to ER Ca^2+^ dysregulation, we examined the magnitude of EPSC changes in the course of repetitive stimulation of LPP or MPP in the absence or presence of thapsigargin (TG; 2 µM, for 30 min), which is a specific inhibitor of SERCA [28], at DG neurons of *PS* cDKO and control mice. We found that the frequency facilitation of EPSCs induced by 6 stimuli applied at a frequency of 20 Hz is diminished in the LPP in slices from control mice after TG treatment (Fig. 2b; F_1, 12_ = 4.77, p = 0.04; two-way ANOVA), but TG has no effect on frequency facilitation in slices from *PS* cDKO mice (Fig. 2b; F_1, 16_ = 0.11, p = 0.75; two-way ANOVA). In the MPP, frequency depression is enhanced following TG treatment (Fig. 2e; F_1, 14_ = 4.72, p = 0.04; two-way ANOVA), but TG has no effect on frequency depression in slices from *PS* cDKO mice (Fig. 2e; F_1, 18_ = 2.41, p = 0.14; two-way ANOVA). Furthermore, EPSC amplitudes in *PS* cDKO neurons are similar to those in control neurons following TG treatment (Fig. 2b and e; LPP: F_1, 14_ = 0.55, p = 0.47; MPP: F_1, 16_ = 0.86, p = 0.37; two-way ANOVA). To compare further the magnitude of EPSC changes in the absence or presence of TG in DG granule neurons of *PS* cDKO and control mice, we assessed the sum of the amplitudes of 6 EPSCs elicited by repetitive stimulation. In the presence of TG, the sum of EPSC amplitudes in DG granule neurons of control mice is significantly reduced in LPP or MPP, respectively (Fig. 2c and f; LPP: Control: 3.06 ± 0.44 nA, Control + TG: 1.61 ± 0.32 nA, p = 0.0012; MPP: Control: 5.15 ± 0.42 nA, Control + TG: 3.38 ± 0.24 nA, p = 0.0002; paired *t*-test). However, TG treatment did not cause further reduction of the integral EPSCs magnitude in DG granule neurons of *PS* cDKO mice (Fig. 2c and f; LPP: *PS* cDKO: 1.70 ± 0.23 nA, *PS* cDKO + TG: 1.64 ± 0.26 nA, p = 0.637; MPP: *PS* cDKO: 3.57 ± 0.19 nA, *PS* cDKO + TG: 3.52 ± 0.18 nA, p = 0.664; paired *t*-test). Taken together, these results show that loss of PS mimics the effects of blockade of SERCA, suggesting that disrupted ER Ca^2+^ homeostasis in *PS* cDKO mice may contribute to the observed impairments in short-term synaptic plasticity.

### Normal spontaneous glutamatergic responses in the perforant pathways in *PS* cDKO mice

To test whether the observed functional changes at the level of synaptic function are associated with abnormal properties of presynaptic cells and/or network activity, we measured spontaneous EPSCs (sEPSCs) and miniature EPSCs (mEPSCs) in *PS* cDKO mice. The frequency of sEPSCs, recorded in DG neurons from *PS* cDKO mice under voltage-clamp conditions in the presence of the GABA_A_ receptor blocker, is not affected (Fig. 3a and b; p = 0.083; unpaired *t*-test), and the sEPSC amplitude also remains unchanged (Fig. 3a and b; p = 0.725; unpaired *t*-test). We further recorded mEPSCs by adding tetrodotoxin (TTX) to the external solution which blocks action potentials, and found that both the frequency and the amplitude of mEPSCs are similar in *PS* cDKO and control neurons (Fig. 3c and d; frequency: p = 0.967, amplitude: p = 0.216; unpaired *t*-test). These results indicate that spontaneous neurotransmitter release is normal in the absence of PS.

### Impaired LTP and NMDAR-mediated responses in the perforant pathways in *PS* cDKO mice

We next examined the effects of PS inactivation on long-term potentiation (LTP) in the LPP and MPP. LTP was induced by five trains of theta burst stimulation (TBS) and potentiation was quantified by measuring the changes in the initial slope of the evoked fEPSPs. We found that LTP is impaired at both LPP and MPP in *PS* cDKO mice. Specifically, the magnitude of LTP measured during the last 10 min post-induction (51–60 min) after TBS in *PS* cDKO mice is significantly reduced (Fig. 4a and e; LPP: 117.3 ± 5.38%, p = 0.023; MPP: 128.7 ± 5.84%, p = 0.022; unpaired *t*-test) relative to control mice (LPP: 137.8% ± 2.93%; MPP: 151.6% ± 6.92%).

To determine whether the observed changes in synaptic plasticity may be caused by the alterations of AMPA receptor function, we examined AMPA receptor-mediated input/output (I/O) curves at the LPP and MPP. We found that AMPA receptor-mediated I/O relationships, obtained by plotting the amplitude of fiber volley (FV) versus the field excitatory postsynaptic potentials (fEPSPs) slope in the presence of blockers of NMDA (50 µM APV) and GABA_A_ receptors (10 µM bicuculline), are similar between *PS* cDKO and control mice at both LPP and MPP (Fig. 4b and f; LPP: p = 0.22; MPP: p = 0.11; linear regression).

Given the importance of NMDA receptors (NMDARs) for the induction of LTP in the studied pathways, we assessed the ratio of the amplitude of NMDAR-mediated EPSCs to the amplitude of AMPAR-mediated EPSCs recorded under whole-cell voltage clamp conditions in DG neurons. We found that the NMDAR/AMPAR ratios for the EPSCs at both LPP and MPP are reduced in *PS* cDKO mice (Fig. 4d and h; LPP: Control: 0.49 ± 0.04, *PS* cDKO: 0.26 ± 0.03, p < 0.0001; MPP: Control: 0.41 ± 0.03, *PS* cDKO: 0.32 ± 0.03, p = 0.028; unpaired *t*-test). Since the efficacy of AMPA receptor-mediated synaptic transmission is unaffected in *PS* cDKO mice, the observed decrease in the NMDAR/AMPAR EPSC amplitude ratio indicates that the function of NMDA receptors is impaired in the absence of PS, possibly contributing to the observed LTP deficits in these mice. Therefore, PS is required for normal long-term synaptic plasticity at the lateral and medial perforant pathways.

## Discussion

We previously reported that PS plays an essential role in regulation of neurotransmitter release, short-term and long-term synaptic plasticity in the hippocampal SC and MF pathways [3, 4, 7–11]. Moreover, we recently demonstrated that genetic ablation of PS selectively in inhibitory neurons results in impaired GABAergic inhibition and hyperactivity of CA1 neurons at the SC synapse of interneuron-specific *PS* cDKO mice [12, 29]. Importantly, dysfunction of the EC-hippocampal network is thought to contribute cognitive impairment observed in AD progression [30]. Specifically, the DG area receives dense EC inputs from the stellate neurons in layer II via perforant pathways, which are profoundly affected in AD [20, 31, 32]. Thus, it was of interest to perform similar electrophysiological analysis to determine functional consequences of PS inactivation at hippocampal LPP and MPP synapses. Our current study demonstrates showed that PS regulates short- and long-term synaptic plasticity and ER Ca^2+^ homeostasis in the perforant pathways.

Similar to our previous findings at hippocampal SC and MF synapses [4, 7–9, 11], we found that short-term synaptic plasticity, such as PPR and frequency facilitation or depression induced by repeated stimulation at higher frequencies, are impaired at hippocampal LPP and MPP synapses in the absence of PS (Fig. 1), indicating an universal requirement of PS for normal short-term synaptic plasticity at hippocampal synaptic connections. To determine whether the observed changes in synaptic plasticity may be caused by abnormal network activity or postsynaptic sensitivity to glutamate, we assayed spontaneous synaptic events. We found no change in the frequency and the amplitude, of both spontaneous and miniature EPSCs in DG granule neurons of *PS* cDKO mice (Fig. 3). These findings are consistent with our earlier findings in CA1 pyramidal neurons [7]. In contrast to the steep Ca^2+^ dependence of evoked synaptic transmission and short-term synaptic plasticity, such as PPR and frequency facilitation/depression, spontaneous neurotransmitter releases, assayed with sEPSCs and mEPSCs, are largely Ca^2+^ independent, and these two modes of neurotransmitter release (evoked *versus* spontaneous) are mechanistically distinct. Thus, the changes in the probability of release at the level of evoked synaptic responses might not be associated with the changes of spontaneous synaptic events.

We previously measured ∆[Ca^2+^]_i_ transients in cell bodies of DG granule neurons, and the cytosolic Ca^2+^ increases were found to be reduced in DG granule neurons of *PS* cDKO mice when induced by higher frequency stimulation (between 5 and 20 Hz) [11]. In the current study, we found that depletion of intracellular Ca^2+^ stores by inhibition of SERCA results in a reduction of the EPSC amplitude in the course of repetitive stimulation of perforant pathways in slices from control mice but not in that from *PS* cDKO mice (Fig. 2). Thus, PS appears to serve as an important regulator of Ca^2+^ homeostasis in hippocampal neurons. Indeed, it has been proposed that PS function as ER Ca^2+^ leak channels [33, 34], as activators of IP_3_R-mediated Ca^2+^ release from the ER [35], or activators of SERCA [36]. Notably, according to our previous measurements of ER Ca^2+^ content ([Ca^2+^]_ER_) in respect to cytosolic Ca^2+^ rise induced by thapsigargin in primary *PS* cDKO hippocampal cultures, the [Ca^2+^]_ER_ is unchanged in *PS* cDKO neurons [9]. These results are supported by other findings showing normal ER Ca^2+^ dynamics in *PS* DKO mouse embryonic fibroblasts (MEFs) [37] as well as unchanged [Ca^2+^]_ER_ in primary *PS1* KO MEFs and immortalized *PS* DKO MEFs following thapsigargin treatments [9]. These results suggest that loss of PS does not affect ER Ca^2+^ concentration in hippocampal neurons, and that the observed defects in Ca^2+^ homeostasis and synaptic function observed in *PS* cDKO neurons are not caused by reduced ER [Ca^2+^].

Although [Ca^2+^]_ER_ was normal in *PS* cDKO hippocampal neurons, PS likely regulates short-term synaptic plasticity via RyR-mediated Ca^2+^ release from the ER [7, 9, 38]. It was shown that blockade of RyRs mimics the effect of SERCA inhibition on synaptic facilitation, whereas blockade of IP_3_Rs has no effect [7]. Moreover, RyR levels are reduced in the hippocampus of *PS* cDKO mice, but the levels of IP_3_Rs and SERCA are unchanged [9]. Consistent with these findings, RyR-mediated Ca^2+^ release from the ER induced by RyR agonists, caffeine or 4-chloro-m-cresol, is reduced in *PS* cDKO hippocampal neurons [9]. Indeed, the presence of RyR and ryanodine-sensitive Ca^2+^ stores in presynaptic terminals of various central synapses has been extensively reported [39–41]. For instance, blockade of RyR and depletion of ER Ca^2+^ stores results in reduction in presynaptic Ca^2+^ transients and paired-pulse facilitation in CA3 pyramidal neurons [42], and caffeine induces presynaptic LTP independent of NMDAR activation or increases of postsynaptic Ca^2+^ [43]. In line with these findings, our previous results showed that reduced RyR expression by shRNA leads to decreases in Ca^2+^-induced Ca^2+^ release and synaptic facilitation, providing further support for the importance of RyR in synaptic function. Specifically, 50% reduction of RyR in *PS* cDKO mice was sufficient to eliminate RyR-mediated synaptic potentiation, and the effect of RyR reduction (~ 50%) by shRNA or PS inactivation on synaptic facilitation is similar to that of RyR blockade by ryanodine or dantrolene [7, 9], suggesting a critical role of RyR in the regulation of synaptic function. These findings suggest that disrupted ER Ca^2+^ homeostasis, possibly by impairment of RyR function, may contribute to the observed synaptic dysfunction in the absence of PS (Fig. 1), and that PS may regulate intracellular Ca^2+^ homeostasis at both LPP and MPP synapses. However, additional studies are needed to address whether Ca^2+^ homeostasis is perturbed in LPP and MPP terminals of the *PS* cDKO mice and whether the observed deficits in short-term synaptic plasticity are contributed by other mechanisms, such as alteration of intracellular Ca^2+^ buffer capacity, as neurotransmitter release is also modulated by intracellular Ca^2+^ buffers in nerve terminals [44–46]. The questions of how PS interacts with RyR in the regulation of intracellular Ca^2+^ release and synaptic facilitation and whether RyRs are physiological substrates of γ-secretase also await future studies.

LTP is a form of synaptic plasticity in both cortical and subcortical regions serving as the cellular substrate of learning and memory [47]. Mechanistically, certain forms of LTP depend on activation of NMDARs, when synaptically-released glutamate causes postsynaptic depolarization, thus promoting activation of NMDARs through depolarization-induced removal of their blockade by external Mg^2+^, and providing intracellular Ca^2+^ influx needed for the induction of LTP [48]. Hence, the changes of NMDAR expression/activation at the postsynaptic side of synapses may account for the alteration in LTP. Our previous studies showed that *PS* cDKO mice exhibit spatial learning and memory impairments and LTP deficits accompanied with reduced NMDAR activation at MF and SC synapses [4, 7–9, 11]. In the present study, similar to our prior findings at MF and SC synapses, the magnitudes of LTP and NMDAR-mediated responses are reduced in lateral and medical perforant pathways of *PS* cDKO mice (Fig. 4). The observation that the changes in the size of NMDAR responses in the LPP are somewhat greater than LTP impairments may suggest that other sources of calcium could contribute to the induction process, possibly blunting the effect of diminished NMDAR activation. Notably, we detected a parallel reduction in synaptic expression levels of NMDAR subunits (NR1 and NR2A) in the cerebral cortex of *PS* cDKO mice at 2 months of age [4], providing a likely molecular basis for the observed impairment of synaptic NMDAR activation after the loss of PS. However, total levels of NMDARs were normal in the cerebral cortex of *PS* cDKO mice, suggesting that the selective reduction in synaptic NMDAR levels may be due to defects in intracellular trafficking or synaptic delivery. Consistent with this notion, our prior study showed a physical interaction between PS1 and NMDARs using cortical lysates [4], suggesting that PS1 forms a stable complex with NMDARs, which may facilitate their proper synaptic delivery or localization. Moreover, calcium/calmodulin-dependent kinase II (CaMKII) is a primary downstream effector of NMDARs in LTP induction, and a previous study demonstrated physical and functional interactions between NMDARs and CaMKII [49]. Specifically, neuronal levels of αCaMKII are regulated by synaptic activity [50] and dendritically-localized αCaMKII plays an essential role in LTP and spatial memory [51]. In *PS* cDKO mice, we previously observed a selective reduction in synaptic and dendritic αCaMKII [4]. Given the role of NMDARs in determining both the synaptic localization and the levels of αCaMKII, the reduction in synaptodendritic αCaMKII may be a consequence of the decreased synaptic activation of NMDARs in *PS* cDKO mice. Furthermore, cAMP response element-binding protein (CREB)-dependent gene expression has been demonstrated to play an important role in the consolidation of LTP and memory [52]. While reduction of CREB-mediated transcription is observed in the cerebral cortex of *PS* cDKO mice [4], CREB-mediated transcription may be regulated indirectly by PS, and attenuation of CREB activation may affect gene expression in *PS* cDKO mice, likely due to reduced neuronal activity in these mutant brains [53]. Despite the fact that PS regulates CREB target gene expression indirectly, it remains possible that the down-regulation of the CREB pathway by loss of PS function plays an important role in the deterioration of normal neuronal function. These findings demonstrate a critical role played by the PS in the regulation of long-term synaptic plasticity in the hippocampus, possibly contributing to learning and memory deficits.

The perforant pathways were thought to be vulnerable in AD pathogenesis due to: (i) synaptic loss observed in the outer molecular layer (OML) of DG and cognitive impairment cases [22, 23]; (ii) the presence of amyloid plaques and neurofibrillary tangles in the EC [54–56] and the OML [18, 54]; and (iii) substantial loss of EC neurons, particularly of layer II [20, 21, 57]. Moreover, selective overexpression of mutant amyloid precursor protein predominantly in layer II/III neurons of the EC resulted in an aberrant excitatory cortico-hippocampal network activity, associated with behavioral abnormalities [58–63]. Thus, the EC and the hippocampus may be involved in various forms of learning and memory that are affected in AD, making the entorhinal-hippocampal projection of significant interest in relation to understanding of the sub-regional progression of AD pathogenesis [64–68]. Despite the importance of PS function at hippocampal synapses [3, 4, 7–12], it remains unclear whether synaptic plasticity in the entorhinal and EC-hippocampal circuits is affected by the loss of PS. Future studies will be needed to determine whether and how PS controls synaptic plasticity in the EC superficial layers. It would be interesting to perform electrophysiological analysis of the EC circuits before and after neurodegenerative alterations to elucidate functional consequences of neurodegeneration and its impact on the EC and EC-hippocampal circuits.

## Data Availability

The datasets generated and/or analyzed during the current study are available from the corresponding author on reasonable request.

## Abbreviations

ACSF: Artificial cerebrospinal fluid
AD: Alzheimer’s disease
AMPA: α-Amino-3-hydroxy-5-methyl-4-isoxazolepropionic acid
CA1: Cornu ammonis 1 area of hippocampus
CA3: Cornu ammonis 3 area of hippocampus
EC: Entorhinal cortex
EPSC: Excitatory postsynaptic current
ER: Endoplasmic reticuli
*PS* cDKO: *Presenilin* conditional double knockout
fEPSP: Field excitatory postsynaptic potential
GABA: Gamma-aminobutyric acid
GABA_A_ receptor: Gamma-aminobutyric acid receptor subtype A
I/O: Input/output
LPP: Lateral perforant pathway
LTP: Long-term potentiation
MF: Mossy fiber
MPP: Medial perforant pathway
mEPSCs: Miniature excitatory postsynaptic currents
NMDA: N-Methyl-d-aspartate
PPF: Paired-pulse facilitation
PPR: Paired-pulse ratio
PS: Presenilins
RyR: Ryanodine receptor
SC: Schaffer collateral
SERCA: Sarcoendoplasmic reticulum Ca^2+^ ATPase
sEPSCs: Spontaneous excitatory postsynaptic currents
TBS: Theta burst stimulation
TG: Thapsigargin
